# The Next Generation of Transcription Factor Binding Site Prediction

**DOI:** 10.1371/journal.pcbi.1003214

**Published:** 2013-09-05

**Authors:** Anthony Mathelier, Wyeth W. Wasserman

**Affiliations:** Centre for Molecular Medicine and Therapeutics at the Child and Family Research Institute, Department of Medical Genetics, University of British Columbia, Vancouver, British Columbia, Canada; Ottawa University, Canada

## Abstract

Finding where transcription factors (TFs) bind to the DNA is of key importance to decipher gene regulation at a transcriptional level. Classically, computational prediction of TF binding sites (TFBSs) is based on basic position weight matrices (PWMs) which quantitatively score binding motifs based on the observed nucleotide patterns in a set of TFBSs for the corresponding TF. Such models make the strong assumption that each nucleotide participates independently in the corresponding DNA-protein interaction and do not account for flexible length motifs. We introduce transcription factor flexible models (TFFMs) to represent TF binding properties. Based on hidden Markov models, TFFMs are flexible, and can model both position interdependence within TFBSs and variable length motifs within a single dedicated framework. The availability of thousands of experimentally validated DNA-TF interaction sequences from ChIP-seq allows for the generation of models that perform as well as PWMs for stereotypical TFs and can improve performance for TFs with flexible binding characteristics. We present a new graphical representation of the motifs that convey properties of position interdependence. TFFMs have been assessed on ChIP-seq data sets coming from the ENCODE project, revealing that they can perform better than both PWMs and the dinucleotide weight matrix extension in discriminating ChIP-seq from background sequences. Under the assumption that ChIP-seq signal values are correlated with the affinity of the TF-DNA binding, we find that TFFM scores correlate with ChIP-seq peak signals. Moreover, using available TF-DNA affinity measurements for the Max TF, we demonstrate that TFFMs constructed from ChIP-seq data correlate with published experimentally measured DNA-binding affinities. Finally, TFFMs allow for the straightforward computation of an integrated TF occupancy score across a sequence. These results demonstrate the capacity of TFFMs to accurately model DNA-protein interactions, while providing a single unified framework suitable for the next generation of TFBS prediction.

## Author Summary

Transcription factors are critical proteins for sequence-specific control of transcriptional regulation. Finding where these proteins bind to DNA is of key importance for global efforts to decipher the complex mechanisms of gene regulation. Greater understanding of the regulation of transcription promises to improve human genetic analysis by specifying critical gene components that have eluded investigators. Classically, computational prediction of transcription factor binding sites (TFBS) is based on models giving weights to each nucleotide at each position. We introduce a novel statistical model for the prediction of TFBS tolerant of a broader range of TFBS configurations than can be conveniently accommodated by existing methods. The new models are designed to address the confounding properties of nucleotide composition, inter-positional sequence dependence and variable lengths (e.g. variable spacing between half-sites) observed in the more comprehensive experimental data now emerging. The new models generate scores consistent with DNA-protein affinities measured experimentally and can be represented graphically, retaining desirable attributes of past methods. It demonstrates the capacity of the new approach to accurately assess DNA-protein interactions. With the rich experimental data generated from chromatin immunoprecipitation experiments, a greater diversity of TFBS properties has emerged that can now be accommodated within a single predictive approach.

## Introduction

Transcription factors (TFs) and their specific binding sites act to modulate the rate of gene transcription. They are central to key biological processes, such as organ development and tissue differentiation, nutrient and environmental stress responses and physiological signals. Delineating specific positions at which TFs bind to DNA is of high importance in deciphering gene regulation at the transcriptional level. Each TF binds a variety of DNA sites with sequence-specific affinity [1]. As TFs bind to DNA in a sequence specific manner, computational methods for motif discrimination have been critically important for the prediction of transcription factor binding sites (TFBSs). Unfortunately, TFBSs are usually short and in most cases TFs are tolerant of sequence variations at many positions of the TFBS. This tolerance for variation impacts the accuracy of genome-scale prediction of TFBSs, as suitable TFBS sequences are found frequently. The accurate prediction of TFBSs is an enduring challenge [2], with ongoing approaches introduced to decrease the high rate of false predictions. In a human cell, it appears that most computationally predicted TFBS are not available for binding, presumably due to chromatin structure and/or local epigenetic properties. Approaches to address the false prediction issue have varied: phylogenetic methods to focus on sequences conserved during evolution [3], using experimentally mapped transcription start site data to focus on promoter proximal regions [4], using histone modification or DNA accessibility data to highlight likely regulatory sequences [5], or focusing on locally dense combinations of motifs [6], [7] defined from TFBS enrichment analysis of co-expressed genes.

Classically, computational prediction of TFBSs is based on models called position weight matrices (PWMs) that reflect the preferred binding motifs associated to corresponding TFs by providing an additive score for any sequence. They approximate the true specificity of a TF and their parameters can be estimated through different methods (see [8] for a review). The basic PWMs make the assumption that each nucleotide within a TFBS participates independently in the corresponding DNA-protein interaction. Basic PWMs can perform well in modeling TFBS properties, but do not account for position interdependencies, that have repeatedly been observed to exist within TFBS motifs using crystal structure analyzes [9], biochemical studies [10]–[12], statistical analyzes of large collections of TFBS [13]–[15], or quantitative analyzes of protein binding microarray (PBM) data [16]–[18]. The latter study based on PBM data has demonstrated that position dependencies are stronger between neighboring positions than others. Incorporating these dinucleotide dependencies into the binding models has been shown to improve predictive accuracy [18], albeit with modest impact in most cases. The recent comparison of several algorithms using PBM binding assays data in [17] also included models considering dinucleotide composition. Recently, a new approach called dinucleotide weight matrices (DWMs) has been developed to extend the basic PWMs by considering the dinucleotide interactions between all pairs of positions within the TFBSs [19].

Moreover, basic PWMs are restricted to the detection of motifs with a fixed length. This constraint has previously led to alternative heuristic approaches for the modeling of TFBS for TFs tolerant of variable widths, such as nuclear receptors [20] and p53 [21], [22]. The analysis of variable spacing in the context of *Escherichia coli* promoter prediction was an early advance in the field, with the spacing between elements addressed with either PFMs [23] or PWMs using logarithms of the probabilities [24] (see [8] for a review). With the growth in TFBS data, early findings of variable TFBS configurations [25], [26] are not unique [27].

Several efforts have explored more flexible models for the prediction of TFBSs. Bayesian hierarchical hidden Markov models (HMMs) have been used to model *cis*-regulatory modules (CRMs) in [28] but TFBSs included in the CRMs are predicted using basic PWMs. In [29], the authors present a Boltzmann chain (i.e. an HMM generalization) to model the competition between DNA-binding factors as TFs which TFBSs are predicted using energy PWMs computed from PBM data. HMMs, which have been widely used in computationally biology for the prediction of protein motifs, have also been applied to the identification of TFBS. Implementations based on HMMs have been made for the detailed study of specific TFs or classes of TFs [20], [25], [30], and in a few cases the methods were generalized as a framework to theoritically analyze TFBS features [11], [31], [32]. The MAPPER software was implemented using HMMs [33], [34], but the approach retained the classic focus on positional independence consistent with the preponderance of data available at the time. The HMMs approach used in [35] allows for flexible length motifs through the use of profile HMMs, with the unconstrained potential for gaps to be incorporated at any position, as previously introduced for protein families [36]. To accommodate dependencies between TFBS positions, [37] introduces a variable-order Bayesian network to model TFBSs, but suggested that greater training data would allow HMM-based approaches to model dependencies at all positions. The growing community interest in the use of HMMs and more advanced models to discriminate TFBS reflects an underlying expectation that emerging data can lead to more effective models.

Recently, a new experimental technique has been developed to study sequences where proteins interact with DNA. This procedure is a combination of chromatin immunoprecipitation and massively parallel sequencing technologies - the well-known ChIP-seq procedure [38]. It gives, with good sensitivity and specificity, DNA sequences to which proteins of interest bind, providing the opportunity to precisely map those binding sites within the genome. Using such data, we can analyze in depth transcription regulation by focusing on DNA sequences that are bound by specific TFs. The availability of thousands of experimentally validated DNA-TF interaction sequences coming from ChIP-seq data and stored in databases such as produced by the ENCODE project [39] allows researchers to develop new approaches for the prediction of specific locations of TFBSs with greater confidence than was previously possible.

We introduce here a novel TFBS model and prediction system based on HMMs, hereafter referred to as TF Flexible Model (TFFM). Building upon previously developed models capturing dinucleotide dependencies and flexible lengths as described previously (see Table S1 for comparison of TFFMs with previous HMM-based TFBS predictions), our approach allows for the capture of these different features within a unique framework. The availability of thousands of ChIP-seq regions for a TF, potentially representing the full diversity of TFBS configurations, motivates the effort to transition to an HMM-based approach to TFBS prediction. The new HMM framework is flexible, supports dinucleotide composition analysis and variable lengths to predict TFBSs. The performance of TFFM was compared to established TFBS prediction methods through analysis of numerous ChIP-seq data sets. Most methods are comparable for TFs exhibiting classic TFBS structures, but TFFMs show distinct performance advantage for the subset of TFs with more diverse binding characteristics. By evaluating the correlation between TFFM scoring with ChIP-seq peak signals and experimentally measured DNA-binding affinities, we found that TFFM scores reflect TF-DNA interactions. Moreover, the probabilistic scheme of the TFFMs allows for a straightforward calculation of a total occupancy score for a DNA region. Researchers may construct and apply the TFFMs through open-source code via an application programming interface at http://cisreg.cmmt.ubc.ca/TFFM/doc/ or directly through our web-based application at http://cisreg.cmmt.ubc.ca/TFFM/. TFFMs build upon the best properties of the established methods, while offering novel capacities within a unified framework. Every TF has its own DNA binding characteristics (position dependencies, spacing, variable flanking regions, occupancy) which can be captured within the unified TFFM-framework.

## Results

### HMM-based framework to predict TFBSs

We present a new HMM-based framework to model and predict TFBSs. HMMs have been extensively used in computational biology to model DNA sequences [36]. They offer a flexible probabilistic method that gives us the opportunity to model TFBSs with their dinucleotide characteristics and that can be extended to take into consideration flexible motifs. In the context of modeling DNA sequences, an HMM is composed of a set of hidden states emitting nucleotides with defined probabilities (corresponding to the set of emission probabilities) and a set of transition probabilities from state to state. HMMs conveniently accommodate large data, deriving an optimal (at least locally) set of probabilities. HMMs can be trained using different well-established algorithms such as the Baum-Welch algorithm [36] or the Viterbi [36] algorithm. We chose the widely used Baum-Welch algorithm since it converges to a local optimum depending on a set of initialized probabilities.

Recent advances in the prediction of TFBSs have incorporated inter-positional properties through the analysis of dinucleotide properties across the sites. To construct models capturing the dinucleotide compositional properties of TFBSs, we implemented two HMM-based approaches.

**Figure 1:**
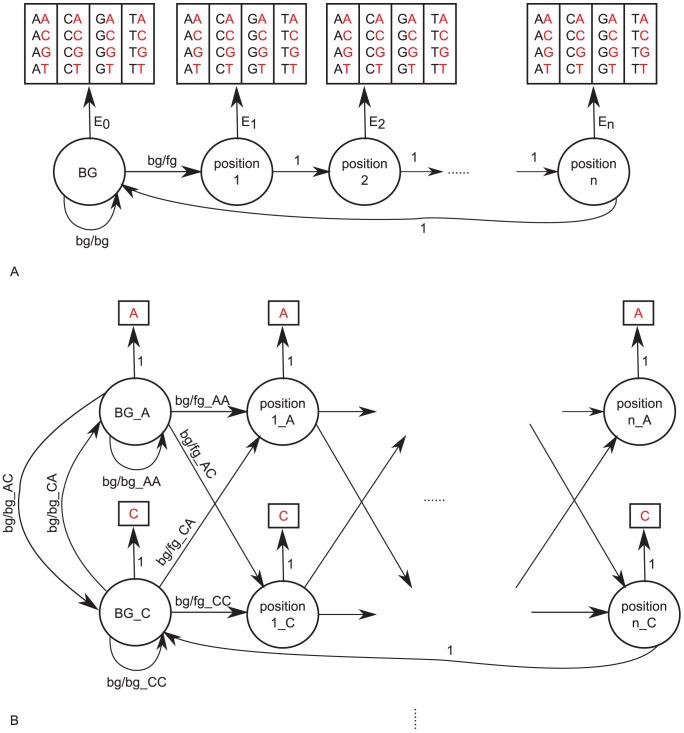
HMM schemas. (A) 1st-order HMM schema used in 1st-order TFFMs where the first state represents the background and the following states the consecutive positions within a TFBS. Each state emits a nucleotide with a probability dependent on the nucleotide emitted previously. (B) HMM schema used in detailed TFFMs where each state in the 1st-order HMM is decomposed into four states (one per nucleotide). Transition probabilities reflects the emission probabilities of the 1st-order HMM. It allows the start of a TFBS depending on the nucleotide emitted by the background states.

Initially, we constructed standard first-order HMMs as TFFMs (denoted later as *1st-order TFFMs*). In such models, each position 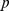 within a TFBS is represented by a state emitting a nucleotide with probabilities dependent on the nucleotide found at the prior position 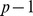 (see Figure 1A). In 1st-order TFFMs, we considered nucleotides surrounding the TFBSs (i.e. nucleotides located before and after a TFBS) by using a specific state modeling the background. The models developed here are similar to the probabilistic dinucleotide PWMs used in [40] to model nucleosomes. However, the 1st-order TFFMs add the capability to capture the properties of the surrounding sequences through the background state, introduce motif length flexibility, and are built upon HMMs as a more dedicated probabilistic framework to model stochastic sequences.

In 1st-order TFFMs, starting a TFBS is given by a unique probability (representing the transition from the background to the TFBS) whatever the nucleotide found in the surrounding sequence. To allow for starting a TFBS depending on the nucleotide emitted in the background state, we implemented a more detailed and descriptive HMM template as TFFMs (denoted later as *detailed TFFMs*) mimicking the theoretical analysis made in [11] (see Figure 1B). The intrinsic different structures of the 1st-order and detailed TFFMs lead to different probabilities for going from the background to the foreground. This is emphasized by the training through the Baum-Welch algorithm which reaches local maximum.

### A graphical representation of the dinucleotide dependencies

**Figure 2:**
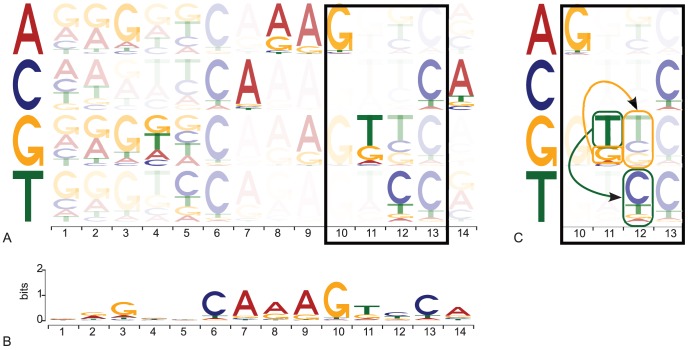
Sequence logo representing a TFFM. (A) Graphical representation of a TFFM constructed for the Hnf4A TF. Each column corresponds to a position within a TFBS. Each row captures the probabilities of each nucleotide to appear depending on the nucleotide found at the previous position. The opacity of a case represents the probability of hitting this case depending on the probability of appearance of the corresponding nucleotide at the previous position (the higher the opacity, the higher the probability). (B) The summary logo compacts all the information to summarize the dense logo in (A). (C) Zooming in on the dense TFFM logo for positions 10 to 13 (corresponding to the box in (A)). We observe that a “C” is more likely to appear at position 12 if nucleotide “T” was found at position 11 whereas a “T” is more likely to appear at position 12 if nucleotide “G” was found at position 11.

By constructing models taking into consideration local dinucleotide dependencies, we aim to better model, characterize, and understand TFBS properties. When trying to analyze and understand a model, a visual representation provides insight into the underlying properties. Basic PWMs for instance can be graphically represented using sequence logos [41] where each position gives the information content obtained for each nucleotide. The greater the height of the letter corresponding to a nucleotide, the higher the information content and higher the probability of getting it at this position. Using HMMs, we can derive the probability of obtaining each nucleotide at each position, allowing for the generation of sequence logos representing the TFBSs modeled using what we call a summary TFFM logo (see Figure 2B). In summary TFFM logos, we use the probability of each nucleotide at each position to compute the corresponding information content represented in the logo. But this graphical representation fails to convey the local dinucleotide dependencies that motivate the work. To tackle this issue, we introduce a new graphical representation (which we call a dense TFFM logo) allowing researchers to perceive the dinucleotide dependencies captured by the model. As the emission of a nucleotide at each position depends on the nucleotide emitted at the previous position, we represent the nucleotide probabilities at position 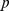 for each possible nucleotide at position 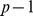. Hence, each column represents a position within a TFBS and each row the nucleotide probabilities found at that position. Each row assumes a specific nucleotide has been emitted by the previous hidden state. The intersection between a column corresponding to position 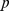 and row corresponding to nucleotide 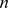 gives the probabilities of getting each nucleotide at position 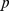 if 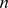 has been seen at position 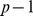 (see Figure 2A). For instance, in Figure 2C, we can observe that a “C” is more likely to appear at position 12 if nucleotide “T” was found at position 11 (green box and arrow) whereas a “T” is more likely to appear at position 12 if nucleotide “G” was present at position 11 (orange box and arrow). In order to highlight the most probable row to be used by the TFFM, we vary the opacity to represent the sequence logo. The higher the probability of getting a nucleotide at position 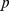, the higher the opacity of the row corresponding to this nucleotide at position 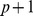. Unfortunately, the current graphical representation does not allow for variable length or spacing of the motif modelled. One could envision introducing the variable length HMMs graphical representation by following the tool specifically developed in [42] for protein families.

### Comparing predictive power of TFFMs with other models on ChIP-seq data

We provide the TFFM-framework to construct TFFMs from ChIP-seq data sets and to predict TFBSs within DNA sequences. When constructing a TFFM from ChIP-seq data, we extract (using MEME [43], [44]) the most over-represented motif out of the top scoring ChIP-seq sequences and use it to initialize the HMM probabilities (see Materials and Methods). Then, the final probabilities are learned using the Baum-Welch algorithm. When predicting TFBSs within DNA sequences using a TFFM, the software gives, at each position within the sequence, the probability of being in a final matching state (corresponding to the last position of a TFBS) in the underlying HMM. These probabilities correspond to posterior probabilities given an HMM and are computed using the well-known forward and backward algorithms [36]. When assessing the predictive power of the models, one can vary a threshold through these output probabilities to compute values of sensitivity and specificity. 1st-order and detailed TFFMs have been constructed using ENCODE [39] ChIP-seq training data sets. The trained TFFMs were used to predict TFBSs within test ChIP-seq data sets by following a 10-fold cross-validation methodology. Finally, the results obtained with TFFMs were compared to the ones obtained from PWMs and DWMs constructed from and applied to the same data.

All ChIP-seq ENCODE data sets from human and mouse (with at least 1800 peaks and a peak max position indicated, i.e. 206 data sets) were used to compare the two types of TFFMs with PWMs and DWMs. Sequences around the peak max positions (50 nucleotides on both sides) were extracted to construct the models and make predictions. The rationale for this is that ChIP-seq peak max positions represent where the maximum amount of ChIP-seq reads map on the genome of reference and TFBSs are expected to be strongly enriched in close proximity to the peak max position [45]. For each data set, the 600 peaks with the highest signal were used to extract the most over-represented motif within the sequences and to initialize the model probabilities. To avoid over-fitting the data when assessing the predictive power of the different models, we used the remaining sequences to construct training and testing data sets following a 10-fold cross-validation approach. Assuming that high quality ChIP-seq data contain at least one true TFBS within each peak region, we considered the hit (matching sequence) with the best score per peak as a TFBS prediction. To assess the level of specificity for each method, we generated background data sets by randomly drawing sequences from mappable regions of the human and mouse genomes by keeping the same %GC composition distribution as in the ChIP-seq testing sequences (see Materials and Methods). Another set of background sequences has been generated from a first-order HMM reflecting the background dinucleotide composition of each ChIP-seq testing data set. Across varying thresholds for TFBS prediction scores for the four different models, we calculate predictive sensitivity and specificity at each threshold value and trace the corresponding receiver operating characteristic (ROC) curves.

**Figure 3:**
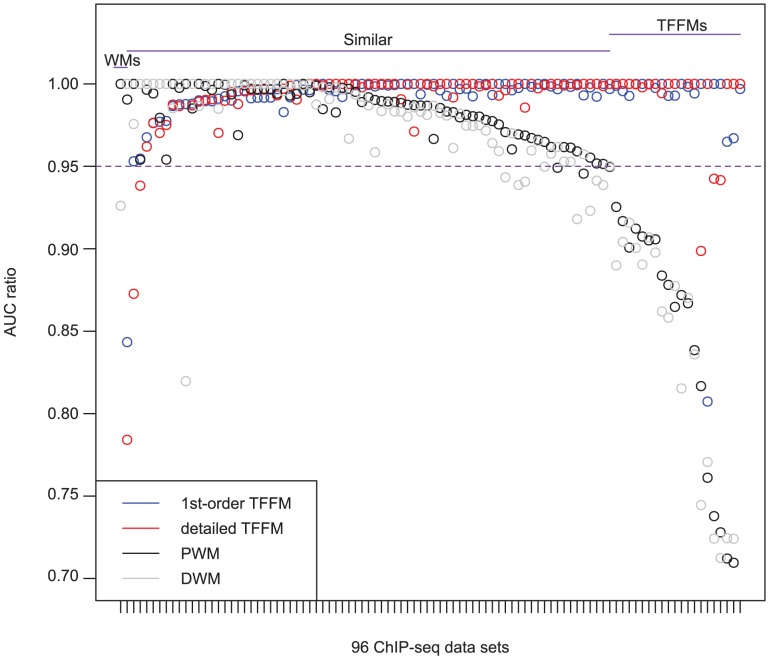
Performance comparison between TFFMs and weight matrices. For the 96 ChIP-seq data sets obtaining an 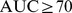
% for at least one method (using a genomic background), the ratio between the AUC value using a specific model and the best AUC obtained is plotted. The four types of models were used (1st-order TFFM, detailed TFFM, PWM, and DWM). By considering a similar performance between two methods when the AUC ratio is 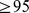
%, we plot at the top of the figure the region where the weight matrices (WMs) best perform, where the TFFMs best perform, and where they are similar. AUC ratios are ranked from the least to the most favourable to the TFFMs.

For each ENCODE ChIP-seq data set, the area under the curves (AUC) for the corresponding ROC curves (for all predictive methods) have been computed. To compare the predictive powers of the different methods, we focus on ChIP-seq data sets for which at least one predictive method achieves an 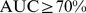 when discriminating ChIP-seq from genomic background sequences (i.e. 96 ChIP-seq data sets). We plot the ratios of performance between the best model and the others on the set of ChIP-seq data for which at least one predictive method is of high quality. In Figure 3, we show the ratio of performance to the best model for ChIP-seq data giving a high quality discriminative performance. When considering a similar performance between models when the ratio of the AUCs is above 95%, we show that the specificity of the binding proteins are captured similarly by using weight matrices (WMs) or TFFMs. Where the performance ratio is below 95%, we can observe an increase in discriminative power in favour of the TFFMs when compared to the WMs (compare the right part of Figure 3 to the left part). When considering the strict difference between respective performance (i.e. when getting strictly higher AUC values), the results indicate that the TFFMs are performing strictly better than both the PWMs and the DWMs in discriminating ChIP-seq peak sequences from background sequences in two thirds of the data sets. Namely, the 1st-order and the detailed TFFMs are performing strictly better than both the PWMs and the DWMs for 63 and 65 data sets, respectively, over the 96 ChIP-seq data sets considered (see Figure 3). Taken together, the TFFMs perform strictly better than WMs in 67 data sets over 96 (70%). Similar results (65 over 94, 69%) are obtained when considering background sequences generated from a first-order HMM (see Figure S1). Explicit AUC values are plotted in Figure S2. We can observe that the TFFMs perform as well as or better than WMs overall on the sets of ChIP-seq data for which at least one predictive method is of high quality when discriminating ChIP-seq sequences from genomic background sequences. See similar results in Figure S3 when considering HMM-generated background sequences.

Statistical significances of the differences in terms of discriminative power between the different methods has been computed for each pair of methods using a Wilcoxon signed rank test [46] assuming the null hypothesis of a symmetric distribution of AUC differences around 0 when two methods perform similarly. Table 1 contains the Benjamini-Hochberg [47] corrected statistical significance of the differences between each one of the predictive methods when considering data sets where at least one method obtains an 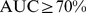. It shows that the performance difference between the two TFFMs is not significant with a 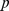-value of 0.528. On the contrary, the difference of performance when comparing both of the TFFMs with the PWMs and the DWMs is statistically significant (see Table 1 where the maximal 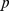-value is equal to 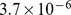). These 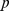-values indicate that the null hypothesis can be rejected. PWMs and DWMs have more comparable behavior since the difference in performance is less statistically significant (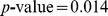, see Table 1). Again, similar results are obtained when considering HMM-generated background sequences (see Table S2).

**Table 1:** Statistical significance for discriminative power differences between the predictive methods.

	1st-order TFFM	detailed TFFM	DWM
**detailed TFFM**	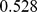	-	-
**DWM**	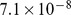	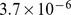	-
**PWM**	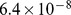	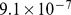	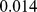

The table contains the Benjamini-Hochberg corrected 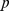-values of the differences (using a Wilcoxon signed rank test) between each pair of methods. 1st-order and detailed TFFMs are likely to perform similarly and so between PWM and DWM. Results are obtained using data sets for which at least one method obtains an 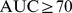
% when discriminating ChIP-seq data from genomic background sequences. Results obtained with HMM-generated background sequences are given in Table S2.

**Figure 4:**
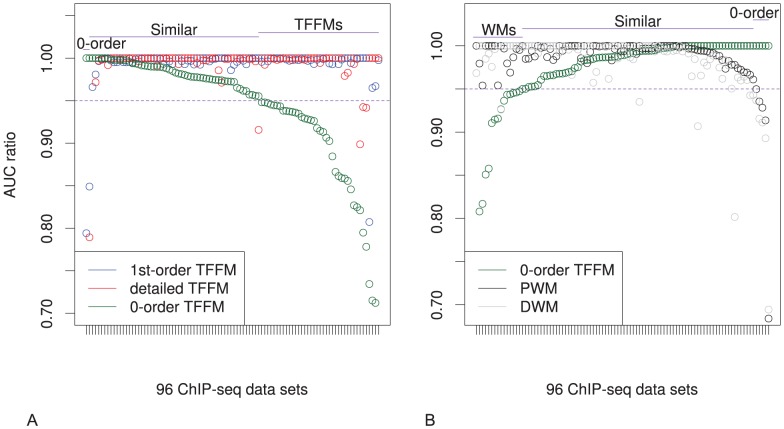
Performances comparison between 0-order TFFMs, other TFFMs, and weight matrices. For the 96 ChIP-seq data sets used in Figure 3 (using genomic background), the ratio between the AUC value using a specific model and the best AUC obtained is plotted. (A) The three types of TFFMs were used (1st-order, detailed, and 0-order TFFMs). AUC ratios are ranked from the least to the most favourable to the 1st-order and detailed TFFMs. We observe that the 1st-order and detailed TFFMs outperform the 0-order TFFMs when discriminating ChIP-seq sequences from genomic background sequences. (B) 0-order TFFMs and WMs were used. AUC ratios are ranked from the least to the most favourable to the 0-order TFFM. We observe that the WMs outperform the 0-order TFFMs when discriminating ChIP-seq sequences from genomic background sequences.

To understand whether the TFFMs perform better than the PWMs because of the model or because of the training method (as both differ), we introduce a 0-order TFFM which is basically a PFM modeled by an HMM and trained using the Baum-Welch algorithm (see Figure S14). We used the 0-order TFFMs to discriminate ChIP-seq sequences from background sequences on the same data sets as previously and computed the corresponding AUCs. In Figure 4A, we observe that the 1st-order and detailed TFFMs outperform the 0-order TFFMs (best AUC of the 1st-order or detailed TFFMs in 90 data sets out of 96, i.e. 94%) emphasizing the need to consider dinucleotide dependencies in the models. In Figure 4B, we compare the 0-order TFFMs to the WMs. The discriminative power of the WMs is higher than the 0-order TFFMs (WMs obtain strictly better AUCs than 0-order TFFMs in 67 data sets out of 96, i.e. 70%, and PWMs perform strictly better than 0-order TFFMs in 63 out of 96 data sets, i.e. 66%) showing that the good performance of the 1st-order and detailed TFFMs does not directly arise from the training methodology since PWMs are performing better, overall, than 0-order TFFMs. Similar results are obtained when using HMM-generated background sequences (see Figure S4).

This analysis shows that the TFFMs perform better than PWMs and DWMs more often, with a statistically significant difference, and lead us to hypothesize that the TFFMs are, overall, better at capturing TFBS features found in the experimental data. To further evaluate this property, we analyzed how TFFM scoring correlates with the biological signal found in ChIP-seq data.

### Correlation between prediction scores and ChIP-seq peak scores

**Figure 5:**
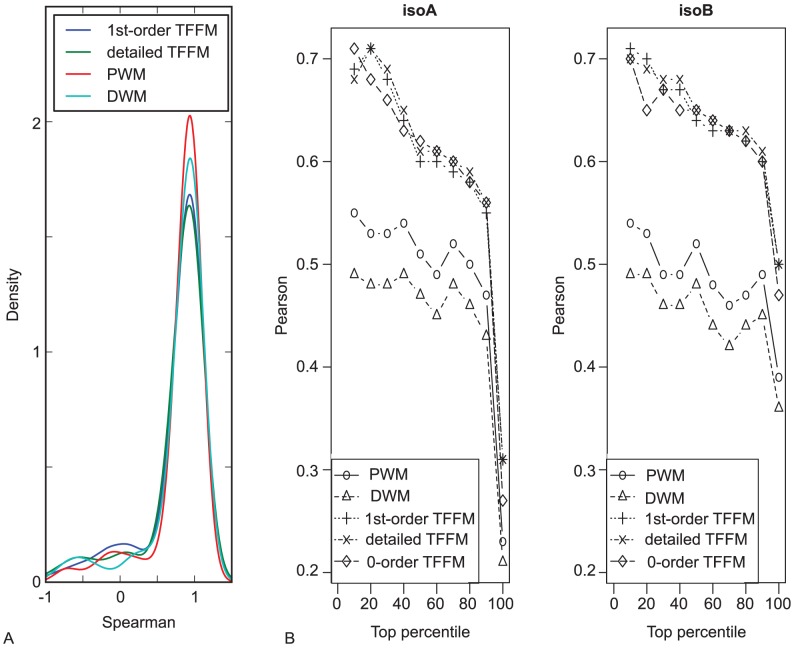
Correlations between prediction scores and ChIP-seq peak scores or binding affinities. (A) ChIP-seq signal values obtained from ENCODE data sets were compared to prediction values obtained with the four different predictive methods. The distribution of Spearman's correlation values from all data sets are given for 1st-order TFFMs, detailed TFFMs, PWMs, and DWMs. An over-representation of Spearman's correlations around 1 (perfect correlation) is found for the four methods. (B) Pearson correlation between scores obtained using the different predictive methods and DNA-binding affinities from [52].

An attractive feature of PWMs is that they can produce scores that are correlated with the energetic binding affinity between a protein and a DNA sequence [48]–[50]. We sought to confirm this property using ChIP-seq data and estimate whether the TFFMs similarly exhibit this capacity. From ChIP-seq experiments, we will assume that the maximum number of reads mapping a peak is representative of the DNA-binding affinity of the corresponding TF to the corresponding DNA sequence (while recognizing the limitations of this assumption). We assess whether the different tested scoring models correlate with the peak signal (corresponding to the level of enrichment for TF-binding within the region) when ranking the peaks with respect to their ChIP-seq signal values compared to ranking with respect to the scores of their best hit. For each data set used in the previous AUC analysis (note that the 600 best peaks are not considered in the 10-fold cross-validation), we extracted the predicted scores associated with each ChIP-seq peak on the foreground testing sets for each of the four predictive methods. ENCODE ChIP-seq data also contain a ChIP-seq signal value associated with each one of the peaks. A potential relationship between prediction scores and peak signal values has been evaluated by separating the ChIP-seq peaks into twenty 5-percentile groups using ChIP-seq peak signal values. Spearman's rank correlation coefficients [51] were computed using the percentiles as the x-axis and the median of prediction scores as the y-axis. The density distribution of Spearman's rank correlation coefficients for each predictive method is given in Figure 5A. The correlation between the prediction scores (for all of the four methods) and the signal value of the corresponding ChIP-seq peaks is mainly located around 1 (with WMs giving slightly better results, see Figure 5A), corresponding to a correlation between the scoring of the methods and ChIP-seq peak signal values. Spearman's rank correlation coefficients indicate that the higher the ChIP-seq peak score, the higher the score we expect to get from the different predictive methods, which is in agreement with the models reflecting the energetic binding affinity between a protein and a DNA sequence. We used 5-percentile groups of sequences (using ChIP-seq peak signal values) to compute Spearman's rank correlation coefficients. The Spearman's rank correlation coefficients computed using all the ChIP-seq peak signal values would result in low correlation values due to the variation of the values from the average trend. Hence, using percentiles gives the general trend that shows the average behaviour of the correlation. We can see from Figure 5A that the trends show a positive correlation. The statistical significance of the trends is assessed by computing the 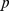-values of getting a regression line (corresponding to the general trend of correlation) for the observed slope. Figure S5 plots the distribution of the computed 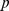-values. We observe that the 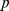-values are, overall, close to 0 and indicate that the average behaviour of the correlation given by a linear regression is far from a horizontal line. These results show that all of the predictive methods have the tendency to correlate with ChIP-seq peak signal values. To further analyze the property of correlation between TFFM scores and binding affinities, we considered experimentally measured binding affinities between a TF and DNA sequences and compared these values to the predictive scores obtained from the different models.

### Correlation between prediction scores and experimental DNA-binding affinities

In the previous section, we hypothesized that the signal values from ENCODE ChIP-seq peaks reflected the affinity of the TF protein to bind to DNA sequences. In [52], Maerkl and Quake measured experimentally the binding affinities of two isoforms of the human Max TF to DNA sequences by testing sequence permutations. The Max TF binds to a core consensus motif of the form CACGTG. As the motif is palindromic, they measured the binding affinities of the Max proteins for all the possible mutated sequences of the first half of the core motif (namely nucleotides 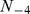 to 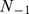 from the consensus sequence, see Figure S6) and conserving the second half of the consensus core binding sites (GTG). We compared these experimental measures to the predictive scores obtained on the Max K562 ChIP-seq data, selected from the available Max ChIP-seq data as it exhibits the highest AUCs in the 10-fold cross-validation experiments for all tested methods. Five different models (0-order TFFM, 1st-order TFFM, detailed TFFM, PWM, and DWM) were constructed from the top 600 peaks for the initialization step and all ChIP-seq sequences were used to train the models. The sequence logo obtained using MEME on the top 600 peaks is given in Figure S6 for reference. We can see that the CACGTG palindromic motif is captured by MEME using the ENCODE ChIP-seq data set. The trained models were then applied to the mutated sequences to get their corresponding predicted scores. Note that the models are trained here on a data set that is independent of the testing data set used in [52].

For each predictive model, we computed the correlation between the predicted scores and the DNA-binding affinity values measured experimentally. Since some mutated sequences can no longer be bound by the Max TF (or with very weak affinity), it is interesting to focus on the sequences to which the TF can actually bind. Hence, we analyzed the correlation between predicted and experimentally measured DNA-binding affinity values by first focusing on the sequences lying in the top 10-percentile affinity values, then the top 20-percentile, and up to including all the sequences using 10-percentile steps. The results of higher interest, corresponding to stronger DNA-binding affinity values, are the top percentiles but all percentiles were computed for completeness. Using such a methodology, we expect the predicted scores obtained from the models to better correlate with high DNA-binding affinity values than with low values. Figure 5B gives the Pearson correlation coefficients with respect to the top percentiles of DNA-binding affinity values for both isoforms of the Max TF. We observe that all the TFFMs (whatever the order) correlate well with the experimental data with a Pearson correlation coefficient over 0.6 up to the top 80-percentiles. Coefficients obtained when considering the top 10- and 20-percentiles (when TF-DNA interaction is the strongest) are around 0.7, showing the high correlation between TFFM scoring and experimental values. In contrast, scores obtained from PWMs and DWMs give Pearson correlation coefficients in the range of 0.45–0.55. Analysis of the Max data indicates that the TFFMs reflect the DNA-binding affinities measured experimentally for the Max TF.

To understand what characteristic(s) the TFFMs are capturing that is not represented by either the PWMs or the DWMs, we examined the DNA sequences obtaining the highest DNA-binding affinity values. We looked at the motifs for which the DNA-binding affinities are the highest by considering the top-scored 25, 50, 75, and 100 sequences (see Figure S7). Figure S8 contains the sequence logos corresponding to the TFFMs and the PWM (no such representation has been made available for the DWM in [19]). One can observe that the TFFMs better capture the C/T pattern at position 7 that is found strongly at position 4 of the top DNA-binding affinity sequences (Figure S7). To a lesser extent, the same can be observed at position 6 with the A/G captured by the TFFMs. These patterns are not captured by the PWM since the CAC is strictly expected at positions 5–7, this coming from the construction and training of the PWMs made from the over-represented motif with a strict CAC. Hence, the PWM does not reflect the needed flexibility captured by the TFFMs through the training step, which is captured by the 0-order TFFM (conceptually the same as a PWM but with a different method of training).

In [52], binding affinity differences between the optimal sequence (with a core CACGTG motif) and the mutated sequences were computed. We performed the same analysis using the predictive scores for the different models in order to compare the results. Table 2 summarizes the comparison in which we observe that the TFFMs perform better than both the PWM and the DWM. Pearson correlation coefficients for TFFMs are 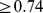 whereas the PWM and the DWM obtain at most a coefficient of 0.66. As a conclusion, these results emphasize the capacity of the TFFM scores to correlate with DNA-binding affinity.

**Table 2:** Pearson correlation coefficients between experimentally measured and predicted changes in affinity correlations.

Isoform	Method	Pearson correlation coefficient
**isoA**	**PWM**	0.65
	**DWM**	0.66
	**0-order TFFM**	**0.76**
	**1st-order TFFM**	**0.74**
	**detailed TFFM**	**0.75**
**isoB**	**PWM**	0.60
	**DWM**	0.61
	**0-order TFFM**	**0.77**
	**1st-order TFFM**	**0.75**
	**detailed TFFM**	**0.75**

The table contains the Pearson correlation coefficients computed between the experimentally measured and predicted changes in DNA-binding affinity between the optimal sequence and mutated ones. Correlations have been computed for both Max TF isoforms and all the predictive methods. Associated 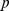-value have been computed for each Pearson correlation coefficient value and are 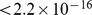.

### Allowing for flexible length motifs using TFFMs

In the previous sections, the TFFMs were used to model TFBSs with fixed length by taking into consideration the dinucleotide composition of the sequences. Another feature of the TFBSs that can be accommodated by TFFMs is flexible length.

A subset of TFs bind to the DNA with different structural conformations, leading to TFBSs of different lengths [25]–[27]. In order to model a binding site with a flexible length, we can use the transition probabilities of the underlying HMMs of the TFFMs. For instance, a 1st-order HMM state corresponding to position 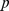 of a TFBS can transition to state at position 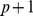 and to state at position 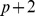 to allow 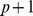 to be omitted for some TFBSs of smaller length. The same applies to HMMs of detailed TFFMs where the probabilities are decomposed for each nucleotide in positions 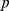 to 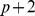. To assess the capacity of the TFFMs to model flexible length TFBSs, we applied the 1st-order and detailed TFFMs to ChIP-seq data corresponding to three TFs with potentially flexible DNA-binding motifs (JunD, STAT4, and STAT6) and compared their discriminative power with fixed length TFFMs, PWMs and DWMs following the cross-validation methodology used previously. We compared the methods to GLAM2 which was first developed to find motifs in proteins with arbitrary insertions and deletions but which can also be applied to DNA sequences [53].

#### The example of the JunD TF

**Figure 6:**
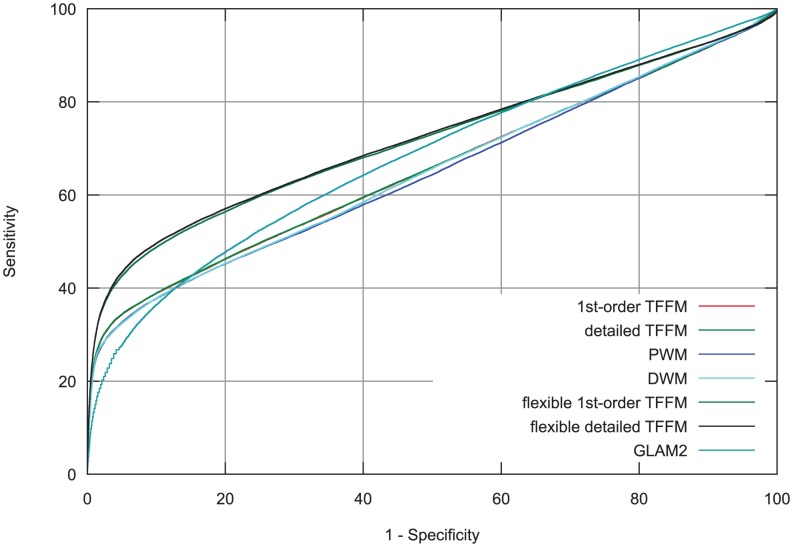
ROC curve analysis of JunD ChIP-seq data. TFFMs allowing a flexible length motif have been compared to PWMs, DWMs, GLAM2, and fixed-length TFFMs. Flexible TFFMs outperform the other models since the corresponding ROC curves are above ROC curves corresponding to other models.

The first TF analyzed is JunD which has been previously shown to bind to motifs of flexible length using protein binding microarrays in mouse [1]. Badis *et al.* showed that JunD can bind to a core motif composed of either TGACGTCA or TGAC/GTCA where C/G stands for C or G. When applying MEME to the whole JunD K562 ENCODE ChIP-seq data set, we observed the enrichment of the two patterns. We used the data set exhibiting the greatest enrichment of the patterns to train and test the different models. We can see from the ROC curves in Figure 6 that introducing a spacer into TFFM for the JunD motif improves the discriminative power of the TFFMs. GLAM2 also performs better than the fixed-length TFFMs, but not as well as the flexible-length TFFMs. The TFFMs using a flexible length outperform the fixed TFFMs, PWMs, DWMs, and GLAM2. Hence, the flexible length TFFMs are able to capture the flexible length motif thanks to the adapted transition probabilities.

#### The examples of STAT4 and STAT6 TFs

**Figure 7:**
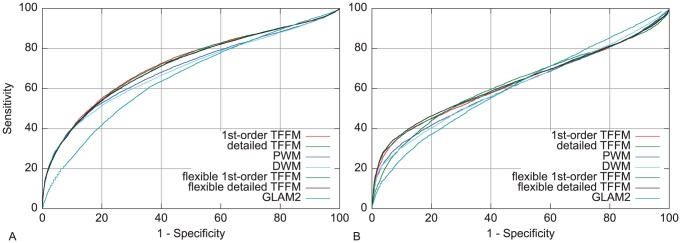
ROC curve analysis of STAT4 and STAT6 ChIP-seq data. TFFMs allowing a flexible length motif have been compared to PWMs, DWMs, GLAM2, and fixed-length TFFMs on STAT4 (A) and STAT6 (B) ChIP-seq data. Flexible TFFMs do not significantly perform better than fixed-length TFFMs. DWMs, PWMs, and GLAM2 produce a lower discriminative power than the TFFMs.

STAT TFs bind with a flexible length motif [25], [26], [54]. STAT4 and STAT6 TFs have been specifically analyzed [54] using ChIP-seq. Even though they were shown to bind to two motifs of different length (STAT4 preferentially binding to a TTCCNGGAA core motif and STAT6 to a TTCCNNGGAA core motif), genes bound by both STAT4 and STAT6 were extracted in different conditions. From such an overlap, one can hypothesize that the TFs can bind to the same motifs when allowing for a flexible spacing between the two halves TTCC and GGAA. To assess this assumption, we constructed 1st-order and detailed TFFMs by allowing for a flexible length in the motif between the two halves. We observe in Figure 7 that introducing a spacer does not improve significantly the AUC of the ROC curves (we observe a small decrease for STAT4 and small increase for STAT6) when compared to the fixed-length TFFMs. All TFFMs perform better than PWMs, DWMs, and GLAM2. Allowing for different spacing within STAT binding motifs and assessing their predictive power has been tested in a previous study without success [27]. This might be due to co-occurrences of binding sites with both 1 nt and 2 nt spacers in a subset of the ChIP-seq data as hypothesized in [27] when looking at STAT5a and STAT5b. This is also supported by [54] (where the ChIP-seq data used are originated) showing that 37% of the genes bound by both STAT4 and STAT6 contain the two types of motifs. The use of TFFMs simplifies the analysis, allowing for a single unified model to capture both motif lengths at no cost for the underlying predictive power.

#### Flexible spacing in the flanking regions of a motif

**Figure 8:**
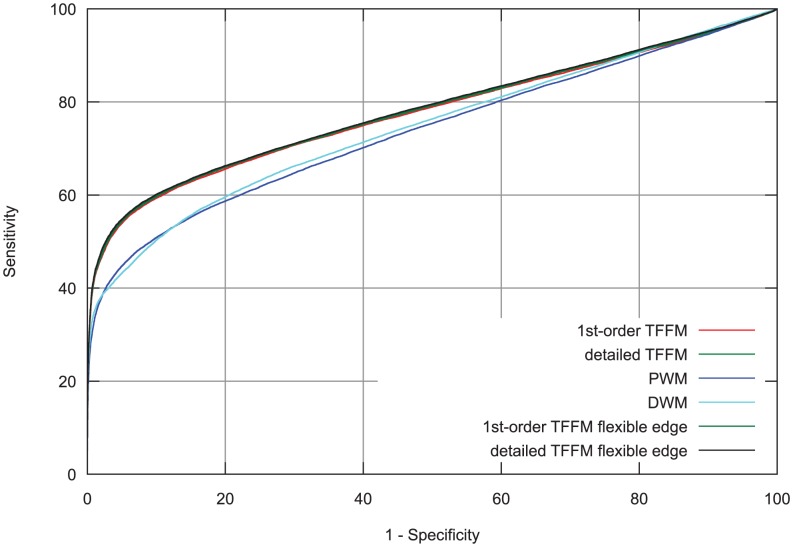
ROC curve analysis of MafK ChIP-seq data. TFFMs allowing a motif with a flexible edge have been compared to PWMs, DWMs, GLAM2, and fixed-length TFFMs. Flexible TFFMs perform slightly better than fixed-length TFFMs and both outperform the other models.

In the previous examples of flexible length binding motifs, we focused on motifs where a spacer was found between the two halves of a core motif. One can also consider motifs containing a flexible edge at the outer edges of a core motif. When analyzing MEME output for ENCODE ChIP-seq data, we observed that a MafK data set showed a weak motif on its edge separated by a 1 nt spacer from the core motif (see Figure S9). We hypothesized a potential flexible edge in the binding motif that could be explained by different biological phenomena as a binding event with or without a co-factor. We assessed this hypothesis by allowing this edge to be present or not when predicting a binding site in the 1st-order and detailed TFFMs. Following the cross-validation methodology used before, we constructed the corresponding ROC curves and compared the results with the previous models. TFFMs outperformed PWMs and DWMs (see Figure 8). We can see from the ROC curves in Figure 8 that introducing a flexible edge gives an improvement to the AUC when compared to the full-length TFFMs. Even though the improvement is not strong, allowing the capture of different types of binding events through a flexible edge gives insight into the binding of MafK. For instance, the flanking portion of motifs might be explained by different conformations of the protein or a potential partnership with a co-factor when binding to the DNA.

### Using TFFMs to compute probabilities of occupancy of a TF

In the previous sections, TFFMs have been used to predict specific TFBS positions. We extended the TFFM-framework to compute an integrated TF occupancy score across a DNA sequence using the TFFM scores. Using the TFFMs, the probability of occupancy (Pocc) of a TF within a defined DNA sequence is obtained by multiplying the TFBS probabilities at each position (see Material and Methods section for details). This is a simpler approach than the physico-chemical models used in tools like GOMER [55] and TRAP [56], and somewhat similar in concept to the approach of OHMM [57] which uses HMMs to predict occupancy of TFs with self-overlapping binding motifs. To observe whether Pocc can improve the discriminative power of the TFFMs, Pocc have been computed from TFFMs and assessed for their capacity to discriminate between ChIP-seq data and background sequences and compared to original TFFMs using the best site per ChIP-seq peak to discriminate between ChIP-seq data and background sequences. When analyzing data sets for which at least one method obtains an 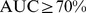 (97 with a genomic background and 100 with an HMM-generated background), we found that using Pocc values improves discrimination as measured by AUC for 62 out of 97 (i.e. 64%) data sets when considering genomic background and 63 out of 100 (i.e. 63%) when considering HMM-generated background (see Figure S14).

## Discussion

In this report, we have introduced a flexible HMM-based framework for TFBS prediction. The new models are demonstrated to perform as well as classic methods for most data, while exhibiting improved performance for a subset of TFs. The new approach retains the desirable attribute of producing scores correlated with the binding energy of TF-DNA interactions. A new graphical representation is introduced to illustrate the properties of the models, complementing the classic and widely used sequence logos. In applications, the TFFM models have been shown to handle variable spacing between half sites, and to allow for the incorporation of flanking sequence properties into TFBS analysis. With a convenient software package and a breadth of opportunities for improvement, TFFMs are a suitable foundation for the next generation of TFBS prediction.

The new TFFM-framework provides an opportunity for researchers to analyze more deeply the features of TF-DNA binding interaction by looking at local dinucleotide dependencies captured by the TFFMs and represented by the new logos. One can see the TFFMs as the probabilistic analog of the energetic BEEML models developed in [18]. Unfortunately, the two modeling approaches cannot be directly compared since they are elaborated from two different types of data sets (PBM data for the BEEML tool [18] and ChIP-seq data for the TFFMs). BEEML software cannot use ChIP-seq data (personal communication with the authors) and the TFFMs have not been developed to consider PBM data information in their current form but both models are able to capture TFBS features with good specificity.

For TFFMs, the greatest utility is in handling the growing subset of TFs with complex binding properties. Such complex binding characteristics of TFs may be decomposed into four categories [1] based on the structure of their corresponding motifs: position interdependence (i.e. the probability of observing a nucleotide at one position is informed by the nucleotide observed at another position), variable width, multiple effects where we can observe a combination of position interdependence and variable length, and alternate recognition interface where bound DNA segments cannot be accounted for using models of either variable length or position interdependence. The models we used in these analyzes aim at addressing the three first categories of TFBS characteristics using only one framework, while the framework provides sufficient flexibility to incorporate the fourth, such as subtle flanking sequence properties.

The TFFM-framework creates new opportunities for innovation in TFBS bioinformatics analysis. Drawing from the initial studies here, it is apparent that refined approaches can be pursued for the identification of TFs capable of binding to motifs of variable width and the analysis of the role of TFBS flanking sequence on TF binding. While the number of cases of TFs tolerant of variable width binding sites has grown with access to high-throughput TFBS data, the TFFM-framework could be extended to enable a comprehensive survey of ChIP-seq data collections to identify additional cases. As observed in the analysis of MafK TFBS flanking sequences, TFFMs are sufficiently flexible to incorporate additional information represented in TFBS proximal sequences. There have been some indications that such sequences may specify interactions with co-factors [58]. TFFMs offer advantages over past methods for the detection of such weak signals with variable positions. It is our plan to expand the TFFM-framework to automatically look for variable-length motifs.

Beyond the analysis of non-canonical TF binding motifs, there is a significant scientific opportunity to develop a new computational approach for the prediction of functionally significant DNA variations within *cis*-regulatory sequences. The global relationship of TFBSs and nucleotide variations is largely unknown [59]. Recent studies have shown extensive genetic variations on human TFBSs often correlated with differences in gene expression [60] and identified TFBSs as genetic determinants of retroviral integration in the human genome [61]. TFFMs have the capacity of modeling the impact of mutations on the TF-DNA binding affinity, as demonstrated for the Max TF. These early results show the promise for using TFFMs to score the impact of nucleotide variations on TF-DNA interactions.

A key to the long-term development and adoption of TFFMs is the access of researchers to both the binding models and the software for their generation. It is our plan to generate a collection of TFFMs trained on ChIP-seq data sets from ENCODE, as well as other sources compiled into the PAZAR repository [62], [63]. Models shall also be incorporated in the next release of the JASPAR collection [64], with a parallel release of PWMs constructed from the same data. Analysis of DNA sequences will be supported through both a web application and a standalone version using already trained TFFMs directly downloadable from JASPAR. For bioinformatics research, we have provided the code of the TFFM package and its documentation (accessible at http://cisreg.cmmt.ubc.ca/TFFM/doc/) to allow others to refine the approaches and make further innovations to broaden the use of TFFMs.

The new TFFMs described in this report are designed to address the confounding properties of position inter-dependencies in site composition and variable lengths observed in experimental data. These two challenges have emerged as an increasing issue with the availability of large-scale ChIP-seq data, which reveals greater complexity of TFBSs than could be observed in the past. The TFFM graphical motif representation conveys properties of position inter-dependence, allowing researchers to visually analyze the features captured by the model. TFFMs have been assessed on human and mouse ChIP-seq data sets coming from ENCODE, revealing a higher discriminative power than established methods. TFFMs produce scores consistent with observed protein-DNA affinities measured experimentally and have the capacity to predict the impact of TF binding site mutations on TF-DNA binding affinities.

The analysis of TFBS is a central challenge in bioinformatics. TFFMs provide a powerful and flexible framework within which a broad range of problems can be addressed. While many motif discrimination methods are available, it is our perception that TFFMs will emerge as a preferred approach for TFBS analysis.

## Materials and Methods

### ChIP-seq data sets

Comparisons between the different predictive methods were done using ChIP-seq data sets from the ENCODE project [39]. We used ChIP-seq data sets from human (hg19 assembly) and mouse (mm9 assembly) containing at least 1800 peaks for which the peak max position is known (i.e. narrowPeak-formatted data), representing 206 ChIP-seq experiments. In limiting to sets with at least 1800 peaks, we ensure that at least two times the number of peaks used to search for an over-represented motif by MEME (top 600 peaks used, see below) will be used during the 10-fold cross-validation procedure as explained below. As DNA-binding events should be located around the peak max area (corresponding to the highest coverage of ChIP-seq reads) of the ChIP-seq peaks [45], we extracted 50 nt on each side of the peak max position. Hence, each peak is composed by 101 nt centered at the peak max position and is associated to a signal value corresponding to the enrichment for TF binding in the region of the peak.

For assessing the performance of TFFMs allowing for flexible length motifs, we used the following ChIP-seq data sets: human ENCODE JunD TF from K562 cells by the University of Chicago, mouse ENCODE MafK TF from Ch12 cells by Stanford University, and STAT4 and STAT6 TFs from [54].

### HMMs

1st-order HMMs used in 1st-order TFFMs are composed of a state modeling the background sequences surrounding TFBSs and one state per position 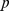 within the TFBSs. The use of a 1st-order HMM allows the model to capture the dinucleotide dependencies through emission probabilities at position 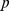 dependent on the nucleotide found at position 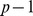 (see Figure 1A). One can move from the background state to the first “matching state” (i.e. the first position within a TFBS) with a defined probability, whatever the nucleotide generated by the background state. Figure 1A gives a representation of the 1st-order HMM template where the first state corresponds to the background state representing the nucleotides surrounding TFBSs. Following states correspond to the matching states where each one corresponds to a position within a TFBS. Each state emits a nucleotide with probabilities dependent on the nucleotide emitted by the previous state. Within matching states, moving from one TFBS position to the next is given by transition probabilities equal to 1. Probabilities are learned using the Baum-Welch algorithm on ChIP-seq sequences, starting from initialized values.

HMMs used in detailed TFFMs decompose each state of the 1st-order HMM with four corresponding states in the detailed HMM, each one emitting a nucleotide (A, C, G, or T) with a probability equal to 1 (see Figure 1B). Dinucleotide dependencies are modeled by four transition probabilities getting out of each state at position 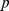 and directing to each state at position 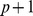 (see Figure 1B).

HMMs used in 0-order TFFMs are constructed with the same set of states as the ones used for the HMMs of the 1st-order TFFMs. The emission probabilities are different since no dependency between positions is captured. Hence, each state is associated to only four emission probabilities for the four nucleotides (see Figure S14).

### Probability of occupancy scores computation

The TFFMs provide, at each position 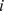 within a DNA sequence, the probability 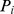 of being in a final matching state (corresponding to the last position of a TFBS). Directly following the spirit of [55], the probability of occupancy (Pocc) of a TF, which TFBSs are modeled by a TFFM, on a DNA sequence of length 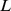 can be computed as:
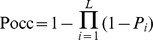
where 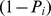 represents the probability of a TF not occupying the DNA sequence at position 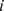.

### Model comparisons

The different model predictive powers were compared using a 10-fold cross-validation methodology on human and mouse ChIP-seq ENCODE data sets.

#### Data sets construction for cross-validation

Given a ChIP-seq data set 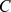, we constructed different sets of sequences that are used to train and test the predictive models. The set 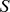 is defined by the top (highest signal-values) 600 ChIP-seq peaks. The remaining sequences (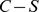) are used to construct the 10 training (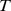) and 10 testing data sets (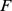) that are used for the 10-fold cross-validation, where 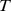 is 9 times larger than 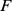.

Ten background data sets (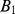) are generated by selecting randomly the same number of sequences from mappable sequences. Mappable regions of the human and mouse genomes have been derived from the ENCODE CrgMappability 36mer track (ftp://hgdownload.cse.ucsc.edu/goldenPath/hg19/encodeDCC/wgEncodeMapability/wgEncodeCrgMapabilityAlign36mer.bigWig). They were generated from contiguous 36mers, and keeping regions greater than 200 bp. These regions were split into 100 bp pieces. The background for each dataset was then drawn randomly by matching %GC composition of the foreground sequences.

Finally, we generate 10 additional background data sets (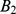) by using a one-state 1st-order HMM constructed from the matching sequences from 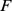.

When predicting TFBSs using a TFFM, input sequences are scored at every positions for both strands. Corresponding scores are the posterior probabilities of being at the final state of the underlying HMM computed by the forward and backward algorithms [36].

#### Model construction for cross-validation

Data sets described in the previous section were used to initialize, train, and test the predictive methods through a cross-validation methodology. The procedure was as follows:

1. Apply MEME [43], [65] on 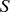 and extract the top over-represented motif (default parameters).
2. Use the motif to initialize the 0-order, 1st-order, and detailed TFFMs (emission probabilities, for the 0-order and 1st-order TFFMs, and transition probabilities, for the detailed TFFM, are derived from nucleotide frequencies at each position of the motif).
3. Train TFFMs on the 10 training sets from 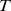.
4. Apply the TFFMs on matching 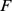 and 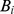 and extract the highest scored hit per sequence.
5. Compute the corresponding ROC curve.
6. Apply MAST [44],[65] on the 10 training sets from 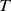 using the motif from MEME (default parameters).
7. Construct corresponding PWMs (i.e. log-odds weight matrices derived from the PFMs [8]) and DWMs using the results from MAST by aligning matched sequences.
8. Apply the PWMs and the DWMs on 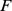 and 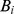 and extract the best hit per sequence.
9. Compute the corresponding ROC curves for each method.

### ROC curves

#### Construction of the ROC curves and AUC computation

For each ChIP-seq data set and predictive model, the best hits from 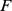 and 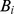 were merged and ranked by their scores (the highest to the lowest). By varying a threshold on the score, we iteratively constructed sets of predicted sequences as TFBSs (i.e. with a score above the threshold). Sequences predicted as TFBSs coming from 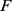 (resp. 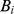) are considered as true (resp. false) positive. On the contrary, true negatives (resp. positive) are sequences coming from 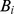 (resp. 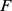) and not predicted as TFBSs (i.e. with a score below the threshold). Each threshold value gives a pair of specificity-sensitivity values used to construct the ROC curve. The area under the ROC curve (AUC) can be computed using the sensitivity as the y-axis and 1 minus the specificity as the x-axis.

#### Computation of the 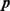-values

The statistical significance of a difference between two predictive methods has been assessed through a 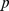-value computation using the two-paired-samples Wilcoxon signed rank test [46] assuming a null hypothesis of symmetric distribution of AUC differences around 0 (function *wilcox.test* of the *stats R* package [66]). 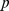-values were corrected using a Benjamini-Hochberg correction for multiple testing [47].

To assess the statistical significance of the trend of correlation between model scores and ChIP-seq signal values, we computed the 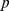-values of getting a regression line (corresponding to the general trend of correlation) with the corresponding slope. The regression lines and the corresponding 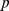-values are obtained through the *lm* function of the *stats R* package [66]. The 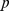-value assesses the probability of getting a slope so far from 0 (horizontal line).

### TFFM logos

The summarized features captured by the TFFMs are represented through a sequence logo similar to the ones used for basic PWMs. To construct the sequence logos, the probability of getting each one of the four nucleotides is computed at each position starting from an equiprobability of A, C, G, and T in the background. Let 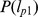 be the probability of letter 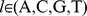 at position 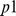. We compute 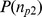 as equal to 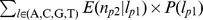 where 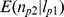 corresponds to the emission probability of the nucleotide 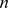 at position 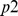 when nucleotide 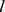 was found at position 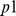. When considering the 1st-order TFFMs, we use the emission probability values whereas the transition probability values give the information for the detailed TFFMs.

The classic sequence logos do not give any information about the dinucleotide dependencies captured by the TFFMs. We introduce a new graphical representation of the TFBSs modelled by the TFFMs that is able to capture this feature (see Figure 2). As for a regular sequence logo, each column corresponds to a position within a TFBS. Each row captures the probabilities of each nucleotide knowing the nucleotide at the previous position (one row per nucleotide A, C, G, and T). It follows the same computation as explained above but considering a specific nucleotide found at the previous position. For instance, the probability of emitting 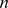 at position 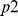 for row A (so A was found at position 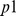) is equal to 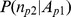. As for the summary logo, the emission probabilities are used for the 1st-order TFFMs whereas the transition probabilities are used for the detailed TFFMs. The height of the letters reflect their probability (the greater the height, the higher the probability). In order to highlight the preferred rows, the opacity of a case (intersection of a row and a column) represents the probability of finding the nucleotide corresponding to this specific row at the previous position of the TFBS (the higher the opacity, the higher the probability).

Given the probabilities of finding each nucleotide at each TFBS position, we compute the information content (IC) of a TFFM by summing the IC of all the positions computed as 
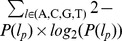
.

### Max experimental DNA-binding affinities

DNA-binding affinities between human Max transcription factor (isoforms A and B) and DNA sequences have been obtained experimentally by using the MITOMI method and reported in [52]. Absolute affinity measures were calculated by varying four nucleotides in the first half of the core binding-motif with the preferred GTG second half-site kept. The changes in the energy between the optimal sequence and mutated ones were also computed in [52] by subtracting the energy associated with the mutated sequences to the energy of the optimal sequence. Both absolute affinity measures and changes in the binding energy were compared to predicted values obtained with the different models.

### Score correlations

#### Prediction scores vs ChIP-seq peak scores

By applying the 10-fold cross-validation methodology to the ChIP-seq data sets (note that the 600 best/top peaks are not considered in the 10-fold cross-validation), we obtained a score for each one of the peaks (corresponding to the score of the best hit per peak). The ENCODE data associate a signal value to each one of the peaks. The signal value is a measure of the enrichment for the overall peak region (usually, average). As the peak scores coming from ENCODE may be unevenly distributed, we computed the median of the distribution of prediction scores for the sequences within each 5-percentile of the peak scores. Hence, each peak score 5-percentile is associated to a predictive score corresponding to the median of their distribution within the percentile. A Spearman's rank correlation coefficient has been used to compare prediction scores and ChIP-seq peak scores for these latter data using the *cor.test* function of the *stats R* package [66].

#### Prediction scores vs experimentally measured DNA-binding affinity

We compared experimental DNA-binding affinities from [52] to prediction scores computed using the different models on all the permuted sequences defined in [52]. The models derived from ChIP-seq data are 11 nt long (see Figure S6) and comparisons focussed on sequences corresponding to positions 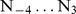 (corresponding to nucleotides NCACGTG in the consensus sequences), see Figure S6. As TFFMs need to incorporate a background context, we generated all the 12 nt long sequences preserving GTG at positions 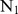, 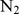, and 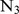. Prediction scores obtained by the models were compared to the experimental values corresponding to the sequence with the same nucleotides at positions 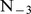, 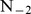, and 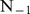. Note that a single value coming from [52] is associated with multiple predictive values for a given model. Results are given in Figure 5B. Experimental scores were iteratively divided using top percentiles (ten by ten) and the Pearson correlation coefficient between experimental values and prediction scores was computed for each set (see Figure 5B).

The detailed methodology used is as follows:

1. Construct all 12 nt-long sequences conserving nucleotides GTG at positions 9, 10, and 11 (a nucleotide A is used at the very beginning for the 1st-order TFFM with no impact on the scores).
2. Construct TFFMs, PWM (i.e. log-odds weight matrices), and DWM using an initialization from the top 600 peaks and trained on the whole ENCODE human Max K562 ChIP-seq data set.
3. Compute the prediction scores on all the sequences using the different methods (only one strand is used for the score computations here).

Score associations between experimental values and results obtained using the above methodology are given in Figure 5B and Table 2.

Starting from the optimal binding site containing CACGTG, we also compared the changes in prediction scores between a mutated site and the optimal sequence with the changes using the experimental DNA-binding affinities for the same sequences. A Pearson correlation coefficient between the two scores is then computed using the function *cor.test* of the *stats R* package [66] (see Table 2).

### Flexible length models

#### JunD TFFMs

The first over-represented motif found by MEME in the JunD data set used for the flexible motif analysis is 14 nt-long with a G/C (G or C) at the centre of the core motif (see position 9 in Figure S10A). The second most over-represented from MEME shows a CG (C followed by a G) at the centre of the core motif (see positions 7–8 in Figure S10B). When initializing the flexible length TFFMs, we added to the 14 nt-long ones the possibility of having either the G/C like pattern at position 9 or a CG like one. Transition probabilities were initialized to allow both motif with 50% probability each. The corresponding TFFMs were then trained on the training data sets following the same 10-fold cross-validation methodology as before.

#### STAT4 and STAT6 TFFMs

To construct a flexible length TFFM modelling STAT4 TFBSs, a gap position has been manually added to the original initialized models between position 5 and 6 (see Figure S11) with equal probabilities for the four different nucleotides. At the initialization step, the probability of using the added gap is set to 50%. Final probabilities have been learned starting from these initialized models using the 10-fold cross-validation methodology on the training sets.

#### STAT6 TFFMs

To construct a flexible length TFFM modelling STAT6 TFBSs, position 5 from the original motif (see Figure S12) was allowed to be ignored. Namely, the transition probability between positions 4 and 5 has been manually set to 50% and a new 50% transition probability between positions 4 and 6 has been added. Hence, the TFFMs were able to model 10 nt- to 11 nt-long motifs. Corresponding initialized TFFMs were trained during the 10-fold cross-validation analysis.

#### MafK TFFMs

Using the original initialized TFFM modelling MafK TFBSs, we allowed the motif to end at position 14 in order to model a flexible edge (see Figure S9). Namely, a 50% transition probability between position 14 and the background state has been added and transition probability between the positions 14 and 15 has been set to 50%. Corresponding initialized TFFMs were trained during the 10-fold cross-validation analysis.

#### GLAM2

GLAM2 [53] has been used on JunD, STAT4, and STAT6 ChIP-seq data sets to predict flexible length motifs. For comparison purposes with the TFFMs, the parameters used were optimized for the search of motifs of the same lengths. Namely, GLAM2 was used to search for 14 nt- to 15 nt-long motifs on the JunD data set, and 11 nt- to 12 nt-long motifs on the STAT4 and STAT6 data sets.

### TFFM-framework

The TFFM-framework is available at http://cisreg.cmmt.ubc.ca/TFFM/doc with a web-based application available at http://cisreg.cmmt.ubc.ca/TFFM/. The software have been implemented in Python using the Biopython tools [67] and the General Hidden Markov Model library [68]. The web-based application has been developed using the Python *cgi_app* (https://pypi.python.org/pypi/cgi_app/1.3) and the Python port of the Template Toolkit (http://template-toolkit.org/python/index.html).

## Supporting Information

Figure S1
**Performances comparison between TFFMs and weight matrices.** For the 94 ChIP-seq data sets obtaining an 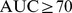
% for at least one method (using the HMM-generated background), the ratio between the AUC value using a specific model and the best AUC obtained is plotted. The four types of models were used (1st-order TFFM, detailed TFFM, PWM, and DWM). By considering a similar performance between two methods when the AUC ratio is 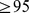
%, we plot at the top of the figure the region where the weight matrices (WMs) best perform, where the TFFMs best perform, and where they are similar. AUC ratios are ranked from the least to the most favorable to the TFFMs.(EPS)

Figure S2
**ROC curves analysis.** Comparison of the AUCs corresponding to the ROC curves analysis of the subgroup of 96 ENCODE ChIP-seq data sets for which at least one predictive method generated an 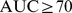
% using the genomic background. The y-axis represents the AUC for the ROC curves corresponding to the different ChIP-seq data sets. The x-axis indicates the number of hits found by MEME in the top 600 sequences of the corresponding ChIP-seq data sets. (A) Each point represent a predictive method applied to a ChIP-seq data set (blue for 1st-order TFFMs, red for detailed TFFMs, black for PWMs, and grey for DWMs). (B) Regression lines obtained from the points in (A) for the four different models. The regression lines corresponding to the TFFMs indicate that higher AUC values have been obtained using TFFMs compared to PWMs and DWMs, overall.(EPS)

Figure S3
**ROC curves analysis.** Comparison of the AUCs corresponding to the ROC curves analysis of the subgroup of 94 ENCODE ChIP-seq data sets for which at least one predictive method generated an 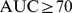
% using the HMM-generated background. The y-axis represents the AUC for the ROC curves corresponding to the different ChIP-seq data sets. The x-axis indicates the number of hits found by MEME in the top 600 sequences of the corresponding ChIP-seq data sets. (A) Each point represent a predictive method applied to a ChIP-seq data set (blue for 1st-order TFFMs, red for detailed TFFMs, black for PWMs, and grey for DWMs). (B) Regression lines obtained from the points in (A) for the four different models. The regression lines corresponding to the TFFMs indicate that higher AUC values have been obtained using TFFMs compared to PWMs and DWMs, overall.(EPS)

Figure S4 **Performances comparison between 0-order TFFMs, other TFFMs, and WMs.** For the 94 ChIP-seq data sets used in Figure S1 (using the HMM-generated background), the ratio between the AUC value using a specific model and the best AUC obtained is plotted. (A) The three types of TFFMs were used (1st-order, detailed, and 0-order TFFMs). AUC ratios are ranked from the least to the most favorable to the 1st-order and detailed TFFMs. We observe that the 1st-order and detailed TFFMs outperform the 0-order TFFMs when discriminating ChIP-seq sequences from HMM-generated background sequences. (B) 0-order TFFMs and WMs were used. AUC ratios are ranked from the least to the most favorable to the 0-order TFFM. We observe that the WMs outperform the 0-order TFFMs when discriminating ChIP-seq sequences from HMM-generated background sequences.
(EPS)

Figure S5 **Statistical significance of the trend of correlation between ChIP-seq peak scores and the different predictive scoring methods.** The statistical significance of the trend plot in Figure 5A is assessed by computing the *p*-value of getting a regression line (between ChIP-seq peak scores and TFFM or WM predicted scores) with an slope that far away from 0. We observe that the p-values are, overall, close to 0 and indicate that the average behaviour of the correlation given by a linear regression is far from an horizontal line.
(EPS)

Figure S6
**Max sequence logo.** Sequence logo corresponding to Max binding sites. Max binds to the DNA with the core site allowing for CACGTG nucleotides at positions 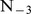, 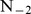, 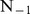, 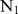, 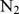, and 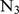.(EPS)

Figure S7 **Sequence logos of the top DNA-binding affinity values.** Sequence logos obtained from the top (A) 25, (B) 50, (C) 75, and (D) 100 scoring sequences using DNA-binding affinities measured experimentally.
(EPS)

Figure S8 **Sequence logos for Max TF.** Sequence logos obtained from the two TFFMs and the PWM after training on all the ChIP-seq data for human Max TF from the K562 cell line. (A) Summary TFFM logo for the 1st-order TFFM. (B) Summary TFFM logo for the detailed TFFM. (C) Sequence logo for the PWM.
(EPS)

Figure S9 **Sequence logo for MafK TF.** Sequence logo from the motif found over-represented with MEME. We observe a weak motif on the edge of the motif, starting at position 15, is separated by a 1 nt spacer from the core motif.
(EPS)

Figure S10 **Sequence logos for JunD TF.** Sequence logos of the top two motifs found over-represented with MEME. (A) The first over-represented motif found by MEME in the JunD data set is 14 nt-long with a G/C (G or C) at the centre of the core motif (see position 9). (B) The second most over-represented from MEME shows a CG (C followed by a G) at the centre of the core motif (see positions 7–8).
(EPS)

Figure S11 **Sequence logos for STAT4 TF.** Sequence logos of the top motif found over-represented with MEME.
(EPS)

Figure S12 **Sequence logos for STAT6 TF.** Sequence logos of the top motif found over-represented with MEME.
(EPS)

Figure S13
**Discriminative power of the probability of occupancy.** Using 1st-order and detailed TFFMs, the ratio between the AUC value obtained from the best site per sequence or the probability of occupancy (Pocc) and the best AUC obtained is plotted. (A) For the 97 ChIP-seq data sets for which at least one method obtains an 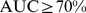 using a genomic background, results from the two kinds of methods (best site per sequence or Pocc) are plotted. AUC ratios are ranked from the least to the most favorable to the usage of the Pocc computation. (B) For the 100 ChIP-seq data sets for which at least one method obtains an 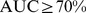 using a HMM-generated background, results from the two kinds of methods (best site per sequence or Pocc) are plotted. AUC ratios are ranked from the least to the most favorable to the usage of the Pocc computation. We observe that using the Pocc computed on the DNA sequences perform better, overall, than using the best score per sequence obtained with the TFFMs on both sets of background sequences.(EPS)

Figure S14 **0-order HMM schema.** 0-order HMM schema used in 0-order TFFMs where the first state represents the background and the following states the consecutive positions within a TFBS. Each state emits a nucleotide with an independent probability for the previously emitted nucleotides.
(EPS)

Table S1 **HMM-based tools for TFBS prediction comparison.** Different HMM-based tools (names and references are given in the first and second columns) have been developed to predict TFBSs. The table compares different features of the tools. The third column indicates whether the models capture the dependencies between TFBS positions. The fourth column indicates whether the implemented HMMs allow for flexible length motifs. The fifth column indicates which tools implement the computation of probability of occupancy (Pocc) of a TF on DNA sequences. The sixth and seventh columns indicate whether the source-code of the tools is freely available for download and whether there is a corresponding online documentation. Finally, the last column indicates the tools for which a web-based application is available.
(PDF)

Table S2
**Statistical significance for discriminative power differences between the predictive methods.** The table contains the Benjamini-Hochberg corrected 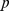-values of the differences (using a Wilcoxon signed rank test) between each pair of methods. 1st-order and detailed TFFMs are likely to perform similarly and so between PWM and DWM. Results are obtained using AUC values when discriminating ChIP-seq data from HMM-generated background sequences.(PDF)
