# Supplementary material for: The Next Generation of Transcription Factor Binding Site Prediction
Source: PLoS Comput Biol. 2013 Sep 5;9(9):e1003214. doi: 10.1371/journal.pcbi.1003214 (PMC3764009; doi:10.1371/journal.pcbi.1003214)
Supplement: Table S1 — HMM-based tools for TFBS prediction comparison. Different HMM-based tools (names and references are given in the first and second columns) have been developed to predict TFBSs. The table compares different features of the tools. The third column indicates whether the models capture the dependencies between TFBS positions. The fourth column indicates whether the implemented HMMs allow for flexible length motifs. The fifth column indicates which tools implement the computation of probability of occupancy (Pocc) of a TF on DNA sequences. The sixth and seventh columns indicate whether the source-code of the tools is freely available for download and whether there is a corresponding online documentation. Finally, the last column indicates the tools for which a web-based application is available. (PDF) [file pcbi.1003214.s015.pdf]

**Method name**	**Reference**	**position interdependence**	**variable length**	**Pocc computation**	**Downloadable open-source code**	**API / online documentation of the source code**	**Web-application**
**N/A**	Raman and Overton [35]	no	yes	no	no	no	no
**N/A**	Mehta, Schwab, and Sengupta [32]	no	no	no	no	no	no
**OHMM**	Drawid et al. [57]	no	no	yes	no	no	no
**MAPPER**	Marinescu, Kohane, and Riva [33]	no	yes	no	no	no	yes
**TFFM**	Mathelier and Wasserman	yes	yes	yes	yes	yes	yes
