# Supplementary material for: The Next Generation of Transcription Factor Binding Site Prediction
Source: PLoS Comput Biol. 2013 Sep 5;9(9):e1003214. doi: 10.1371/journal.pcbi.1003214 (PMC3764009; doi:10.1371/journal.pcbi.1003214)
Supplement: Table S2 — Statistical significance for discriminative power differences between the predictive methods. The table contains the Benjamini-Hochberg corrected -values of the differences (using a Wilcoxon signed rank test) between each pair of methods. 1st-order and detailed TFFMs are likely to perform similarly and so between PWM and DWM. Results are obtained using AUC values when discriminating ChIP-seq data from HMM-generated background sequences. (PDF) [file pcbi.1003214.s016.pdf]

	**1st-order TFFM**	**detailed TFFM**	**DWM**
**detailed TFFM**	0.04031	-	-
**DWM**	3×10^{-5}	2.4×10^{-4}	-
**PWM**	1.5×10^{-7}	6.4×10^{-6}	0.09043
